# A nomogram for predicting cause-specific mortality among patients with cecal carcinoma: a study based on SEER database

**DOI:** 10.1186/s12876-023-02802-7

**Published:** 2023-05-23

**Authors:** Qianru Zhou, Yan Zhan, Jipeng Guo

**Affiliations:** 1 The Central Hospital of Wuhan, Tongji Medical College, Huazhong University of Science and Technology, Wuhan, 430014 China; 2 Wuhan Central Hospital, No. 26, Shengli Street, Jiang’an District, Wuhan, China

**Keywords:** CC, Competing risk model, SEER database, Cause-specific mortality

## Abstract

**Objective:**

Classical Cox proportional hazard models tend to overestimate the event probability in a competing risk setup. Due to the lack of quantitative evaluation of competitive risk data for colon cancer (CC), the present study aims to evaluate the probability of CC-specific death and construct a nomogram to quantify survival differences among CC patients.

**Methods:**

Data on patients diagnosed with CC between 2010 and 2015 were collected from the Surveillance, Epidemiology, and End Results Program (SEER) database. Patients were divided into a training dataset for the establishment of the model and a validation dataset to evaluate the performance the model at a ratio of 7:3. To evaluate the ability of multiple variables to predict cause-specific death in CC patients, univariate and multivariate analyses with Fine-Gray models were performed to screen the predictors of cause-specific death, and a nomogram for predicting cause-specific mortality was constructed. Then, the receiver operating characteristic (ROC) curve and the calibration curve were plotted to evaluate the prognostic performance of the nomogram.

**Results:**

The dataset was randomly divided into a training (*n* = 16,655) dataset and a validation (*n* = 7,139) dataset at a ratio of 7:3. In the training dataset, variables including pathological subtypes of tumors, pathological grading (degree of differentiation), AJCC staging, T-staging, surgical type, lymph node surgery, chemotherapy, tumor deposits, lymph node metastasis, liver metastasis, and lung metastasis were identified as independent risk factors for cause-specific death of CC patients. Among these factors, the AJCC stage had the strongest predictive ability, and these features were used to construct the final model. In the training dataset, the consistency index (C-index) of the model was 0.848, and the areas under the receiver operating characteristic curve (AUC) at 1, 3, and 5 years was 0.852, 0.861, and 0.856, respectively. In the validation dataset, the C-index of the model was 0.847, and the AUC at 1 year, 3 years, and 5 years was 0.841, 0.862, and 0.852, respectively, indicating that this nomogram had an excellent and robust predictive performance.

**Conclusion:**

This study can help clinical doctors make better clinical decisions and provide better support for patients with CC.

## Introduction

Colorectal cancer proves to be one of the most common cancers and leading causes of cancer-related death worldwide [1]. The morbidity and mortality caused by colorectal cancer among men are higher than those among women [2, 3]. It is reported that approximately 20% of colorectal cancer arises in the cecum [4]. Patients with CC are more likely to be diagnosed with a more advanced stage due to the non-specific obscure symptoms at an early stage [5]. Compared with patients with other types of colorectal cancers, the prognosis of patients with CC is often poorer. In addition, epidemiological studies have revealed a declining incidence of left colon cancer (LCC) and an increased incidence of right colon cancer (RCC) [6, 7], in which the incidence of primary colon cancer has the highest increase. RCC is more likely to be exophytic than LCC and lower overall survival is seen in patients with RCC [8–10]. The two most prevalent types of RCC are CC and ACC. Both of them are believed to form in the midgut, but differences may emerge between the adenocarcinomas of them due to different origins and development processes. A recent study demonstrated that the prognosis of CC patients was worse than that of ACC patients, indicating that CC patients have to carry a heavier burden [11]. Therefore, identifying the predictor variables that affect the prognosis of CC patients is significant to help clinicians to formulate more appropriate personalized strategies for the diagnosis and treatment of patients.

It is common practice to use Cox proportional hazards models and the Kaplan–Meier estimator to determine prognostic factors for CC patients [12–14]. Considering the significance of personalized treatment, it is necessary to identify cancer-related and non-cancer-related factors affecting patient mortality, as non-cancer factors such as suicide and traffic accidents other than cancers are often reported to cause death [15, 16]. In studies investigating prognostic factors for cancer patients, non-cancer factors responsible for the mortality of patients are generally considered competitive events in the presence of which multiple endpoints coexist and compete with one another to generate competing risk data [17–19]. Due to the existence of competing risks, survival analysis targeting a single endpoint of interest will yield biased results [20–22]. In terms of traditional approaches to survival analysis such as standard survival analysis using Cox proportional hazard model, the probability of one event over time is estimated, in which the occurrence of one type of death will prevent the occurrence of the death from other factors. However, in our study, CC-specific death and death from other factors are competing events. In this scenario, the use of Cox model to analyze competing event data tends to bias the results, that is, overestimate the mortality in CC patients. However, a competing risk model can be used to investigate the predictive variables that affect the prognosis of patients with CC. What’s more, the comparison of results from competing risk models and those from conventional methods for survival analysis helps to illustrate the actual effects of multiple predictors and presents more accurate estimates of outcome probabilities.

The present study was conducted to construct a nomogram based on clinical data to predict cause-specific mortality for CC patients. Clinical information was obtained from the SEER database. The results of survival analysis to estimate the probability of cause-specific mortality over time using a Cox proportional hazards model vs a competing risk model were compared, and it was found that the Cox model produced inaccurate estimates, that is, underestimated the probability of survival in CC patients. Therefore, a competing risk model was constructed to reduce the likelihood of biased estimates. CC was identified as the clinical outcome, and non-cancer causes of death as competing risks to more accurately predict the cause-specific mortality for patients with CC.

## Materials and methods

### Data collection

SEER * Stat software (version 8.4.1) was used to extract the data in the SEER database (https://seer.cancer.gov/) about patients who were diagnosed with CC between 2010 and 2015. The diagnosis of CC is based on the third edition of the International Classification of Diseases Oncology (ICD-O-3). We collected demographic information such as age, marital status, gender, and race of patients, as well as clinical information related to tumor pathology, tumor differentiation level, AJCC staging (7th edition), T staging, N staging, surgical procedures, lymph node dissection, distant metastasis site surgery, radiotherapy, chemotherapy, tumor deposits, lymph node metastasis, bone metastasis, brain metastasis, liver metastasis, lung metastasis, tumor size, and patient survival time and status. Data were excluded if (1) patients had a survival time of less than one month were excluded, (2) patients were younger than 18 or older than 100 years old and information patients were involved in missing information; (3) data involving unknown causes of death (COD). As for continuous variables, patients were split into three groups by age: < 40 years old, 40 to 60 years old, and > 60 years old. They were divided into three groups by tumor size: < 30 mm, 30 to 50 mm, and > 50 mm. In the present study, CC-specific death and death from factors except CC compete with each other to deliver the event of interest.

### Statistical analysis

The demographic and clinical characteristics of patients in the training and validation dataset were analyzed. The Chi-square test was utilized for describing distributional differences between two datasets. Multiple variables were screened by univariate and multivariate analysis using the Fine and Gray regression model. Variables with *P* < 0.05 in the univariate analysis were then included in multivariate analysis, in which factors with *P* < 0.05 were then used to construct the final competing risk model and nomogram. We calculated the concordance index (C-index) of the final model to evaluate its performance. Calibration plots and receiver operating characteristic (ROC) curves were used to compare predicted and observed probabilities to analyze the performance of the model. We performed statistical analyses using software R version 4.1.2 (https://www.r-project.org/). The tableone package (version 0.13.2) was employed for data description and riskRegression (2021.10.10) for conducting Fine and Gray regression analysis and establishing the competing risk model. Two-tail P value less than 0.05 was the threshold of statistical significance.

## Results

### Patient characteristics

Data on 23,794 patients were extracted. These patients were split into a training set (*N* = 16,655; 70%) and a validation set (*N* = 7,139; 30%). Among them, 10,992 patients died during the follow-up period (7,013 dying from CC and 3,979 dying from other causes). Patients dying from non-cancer-related causes accounted for 36.20% of the total deaths. Among all patients, those over 60 years old accounted for 95.85% of the total (*n* = 22,807); 13,083 patients (54.98%) were female, and 10,711 (45.02%) were male. Most patients (*n* = 14,173; 59.57%) had tumors with a size of fewer than three centimeters, and 22.98% (*n* = 5469), 30.25% (*n* = 30.25), 32.2% (*n* = 7662), and 14.57% (*n* = 3466) have stage I, II, III, and IV tumors, respectively. No significant difference was observed regarding the follow-up data in the training and the validation data set (Table 1).

**Table 1:** Basic characteristics of patients

Factors	Define	Train(*N* = 16,655)	Test(*N* = 7139)	All(*N* = 23,794)
Age	< 40	37(0.22)	17(0.24)	54(0.23)
	40 ~ 60	656(3.94)	277(3.88)	933(3.92)
	60 ~	15,962(95.84)	6845(95.88)	22,807(95.85)
Marriage	Married	8862(53.21)	3777(52.91)	12,639(53.12)
	Divorced	2452(14.72)	989(13.85)	3441(14.46)
	Single	3548(21.3)	1546(21.66)	5094(21.41)
	Other	1793(10.77)	827(11.58)	2620(11.01)
Race	White	13,563(81.44)	5828(81.64)	19,391(81.5)
	Black	2121(12.73)	901(12.62)	3022(12.7)
	Other	971(5.83)	410(5.74)	1381(5.8)
Sex	Female	9168(55.05)	3915(54.84)	13,083(54.98)
	Male	7487(44.95)	3224(45.16)	10,711(45.02)
Behav	Behav1	10,993(66)	4676(65.5)	15,669(65.85)
	Behav2	1880(11.29)	744(10.42)	2624(11.03)
	Behav3	1648(9.89)	745(10.44)	2393(10.06)
	Behav4	928(5.57)	455(6.37)	1383(5.81)
	Other	1206(7.24)	519(7.27)	1725(7.25)
Grade	I	1311(7.87)	532(7.45)	1843(7.75)
	II	11,230(67.43)	4808(67.35)	16,038(67.4)
	III	3324(19.96)	1441(20.18)	4765(20.03)
	IV	790(4.74)	358(5.01)	1148(4.82)
Stage	I	3879(23.29)	1590(22.27)	5469(22.98)
	II	5018(30.13)	2179(30.52)	7197(30.25)
	III	5342(32.07)	2320(32.5)	7662(32.2)
	IV	2416(14.51)	1050(14.71)	3466(14.57)
Tstage	T1	1874(11.25)	763(10.69)	2637(11.08)
	T2	2808(16.86)	1194(16.73)	4002(16.82)
	T3	8313(49.91)	3618(50.68)	11,931(50.14)
	T4	3660(21.98)	1564(21.91)	5224(21.96)
Nstage	N0	9211(55.3)	3915(54.84)	13,126(55.17)
	N1	4012(24.09)	1772(24.82)	5784(24.31)
	N2	3432(20.61)	1452(20.34)	4884(20.53)
Surgery	Surg1	13,275(79.71)	5751(80.56)	19,026(79.96)
	Surg2	2777(16.67)	1122(15.72)	3899(16.39)
	Surg3	191(1.15)	88(1.23)	279(1.17)
	Surg4	412(2.47)	178(2.49)	590(2.48)
LNSur	4 ~	15,845(95.14)	6838(95.78)	22,683(95.33)
	1 ~ 3	490(2.94)	172(2.41)	662(2.78)
	None	320(1.92)	129(1.81)	449(1.89)
SurgOth	None	15,446(92.74)	6621(92.74)	22,067(92.74)
	Yes	1209(7.26)	518(7.26)	1727(7.26)
Radiation	None	16,416(98.56)	7020(98.33)	23,436(98.5)
	Yes	239(1.44)	119(1.67)	358(1.5)
Chemotherapy	None	5657(33.97)	2386(33.42)	8043(33.8)
	Yes	10,998(66.03)	4753(66.58)	15,751(66.2)
Deposits	None	4339(26.05)	1892(26.5)	6231(26.19)
	Yes	12,316(73.95)	5247(73.5)	17,563(73.81)
LnPositive	No	9179(55.11)	3954(55.39)	13,133(55.19)
	1 ~ 3 Positive	3705(22.25)	1586(22.22)	5291(22.24)
	4 ~ 6 Positive	1556(9.34)	676(9.47)	2232(9.38)
	> 7 Positive	2215(13.3)	923(12.93)	3138(13.19)
Bone	None	16,585(99.58)	7108(99.57)	23,693(99.58)
	Yes	70(0.42)	31(0.43)	101(0.42)
Brain	None	16,636(99.89)	7133(99.92)	23,769(99.89)
	Yes	19(0.11)	6(0.08)	25(0.11)
Liver	None	15,064(90.45)	6441(90.22)	21,505(90.38)
	Yes	1591(9.55)	698(9.78)	2289(9.62)
Lung	None	16,314(97.95)	6973(97.67)	23,287(97.87)
	Yes	341(2.05)	166(2.33)	507(2.13)
Size	< 3 cm	9941(59.69)	4232(59.28)	14,173(59.57)
	3 ~ 5 cm	2937(17.63)	1289(18.06)	4226(17.76)
	> 5 cm	3777(22.68)	1618(22.66)	5395(22.67)

(1) Disease classification: Behav1-adenocarcinoma, NOS; Behav2-adenocarcinoma in tubulovillous adenoma; Behav3-mucinous adenocarcinoma; Behav4-adenocarcinoma in adenomatous poly; (2) Operation types: Sugery1-subtotal colectomy/hemicolectomy (partial colectomy but less than total colectomy, right or left colectomy (resection of left or right colon and partial transverse colon), or additional resection of other organs); Surgery2-partial colectomy but less than hemicolectomy or additional resection of adjacent organs; Surgery3 -no operation or tumor destruction, Surgery4-other extended operations

### Variable selection

**Fig. 1:**
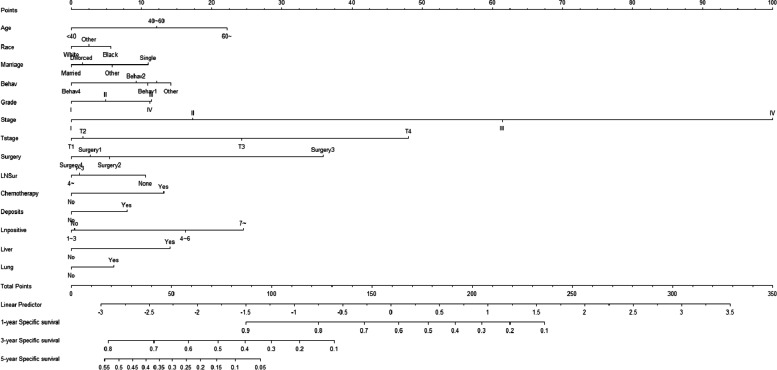
The competing risk nomogram for predicting 1-year, 3-year, 5-year cause-specific survival probability of cecal carcinoma

According to the results of univariate analysis, risk factors with *P* < 0.05 included pathological type of tumor, bone metastasis, brain metastasis, liver metastasis, lung metastasis, tumor grade (degree of differentiation), lymph node dissection, T stage, N stage, M stage, lymph nodes-positive, tumor size, AJCC stage, etc. These variables were then included in the multivariate analysis and the results showed that the following factors affected the survival of patients: age (< 40 years as a reference, 40 to 60: HR = 1.638, 95% CI [0.803, 3.342]; > 60: HR = 2.189, 95% CI [1.089, 4.399]), race (White as a reference, Black: HR = 1.162, 95% CI [1.068, 1.264]), marital (being married as a reference; divorced: HR = 1.014, 95% CI [0.93, 1.106]; single: HR = 1.304, 95% CI [1.201, 1.415]), pathological type of tumor (Behav1 as a reference, Behav2: HR = 0.945, 95% CI [0.841, 1.063], Behav3: HR = 1.009, 95% CI [0.921, 1.106]; Behav4: HR = 0.773, 95% CI [0.652, 0.916]), tumor stage (Stage I as a reference, Grade 1 as a reference; Grade 2: HR = 1.094, 95% CI [0.958, 1.249]; Grade 3: HR = 1.338, 95% CI [1.161, 1.542]; Grade 4: HR = 1.283, 95% CI [1.071, 1.537]), AJJC Stage (Stage I as a reference, Stage II: HR = 1.419, 95% CI [1.161, 1.734]; Stage III: HR = 3.68, 95% CI [2.914, 4.64]; Stage IV: HR = 9.704, 95% CI [7.634, 12.335]), T stage (T1 as a reference, T2: HR = 1.14, 95% CI [0.942, 1.378; T3: HR = 1.759, 95% CI [1.445, 2.141]; T4: HR = 3.029, 95% CI [2.48, 3.7]), type of surgeries(Surg1 as a reference, Surg2: HR = 1.047, 95% CI [0.964, 1.136]; Surg3: HR = 2.252, 95% CI [1.56, 3.251]; Surg4: HR = 0.942, 95% CI [0.789, 1.124]), lymph node dissection (> 4 as a reference;1 ~ 3: HR = 0.927, 95% CI [0.705, 1.219]), chemotherapy(None as a reference, Yes:HR = 1.554, 95% CI [1.444, 1.672]), deposits(None as a reference, Yes: HR = 1.179, 95%CI [1.093, 1.272]), lymph nodes-positive (No as a reference, 1 ~ 3: HR = 1.311, 95% CI [1.044, 1.646]; 4 ~ 6:HR = 1.714, 95% CI [1.276, 2.3]; > 7: HR = 2.234, 95% CI [1.681, 2.971]), liver metastasis (None as a reference; Yes: HR = 1.446, 95% CI [1.302, 1.605), lung metastasis (None as a reference; Yes: HR = 1.206, 95%CI [1.064, 1.367]) (Table 2). These variables were used to construct a competing risk model to estimate the probability of cause-specific mortality in CC patients at 1, 3, and 5 years (Fig. 1).

**Table 2:** Univariate and multivariate analysis

Factors	Define	Univariate analysis		Multivariate analysis	
		HR (95%CI)	Z(P)	HR (95%CI)	Z(P)
Age	< 40	Ref	NA	Ref	NA
	40 ~ 60	1.346(0.648 ~ 2.795)	0.796(0.43)	1.638(0.803 ~ 3.342)	1.356(0.17)
	60 ~	1.653(0.808 ~ 1.106)	1.375(0.17)	2.189(1.089 ~ 4.399)	2.2(0.03)
Marriage	Married	Ref	NA	Ref	NA
	Divorced	1.133(1.044 ~ 1.230)	3.004(< 0.01)	1.014(0.93 ~ 1.106)	0.323(0.75)
	Single	1.214(1.130 ~ 1.303)	5.342(< 0.01)	1.304(1.201 ~ 1.415)	6.339(< 0.01)
	Other	1.224(1.119 ~ 1.339)	4.422(< 0.01)	1.108(1.006 ~ 1.22)	2.081(0.04)
Race	White	Ref	NA	Ref	NA
	Black	1.174(1.084 ~ 1.270)	3.958(< 0.01)	1.162(1.068 ~ 1.264)	3.492(< 0.01)
	Other	0.978(0.865 ~ 1.106)	-0.355(0.72)	1.017(0.895 ~ 1.156)	0.262(0.79)
Sex	Female	Ref	NA	Ref	NA
	Male	1.007(0.952 ~ 1.064)	0.231(0.82)	1.048(0.985 ~ 1.115)	1.473(0.14)
Behav	Behav1	Ref	NA	Ref	NA
	Behav2	0.542(0.486 ~ 0.604)	-11.059(< 0.01)	0.945(0.841 ~ 1.063)	-0.944(0.35)
	Behav3	1.028(0.938 ~ 1.127)	0.599(0.55)	1.009(0.921 ~ 1.106)	0.201(0.84)
	Behav4	0.478(0.407 ~ 0.562)	-8.960(< 0.01)	0.773(0.652 ~ 0.916)	-2.967(< 0.01)
	Other	1.292(1.168 ~ 1.429)	4.992(< 0.01)	1.119(0.995 ~ 1.259)	1.883(0.06)
Grade	I	Ref	NA	Ref	NA
	II	1.678(1.464 ~ 1.922)	7.456(< 0.01)	1.094(0.958 ~ 1.249)	1.32(0.19)
	III	3.228(2.802 ~ 3.792)	16.221(< 0.01)	1.338(1.161 ~ 1.542)	4.026(< 0.01)
	IV	3.459(2.917 ~ 4.102)	14.267(< 0.01)	1.283(1.071 ~ 1.537)	2.702(0.01)
Stage	I	Ref	NA	Ref	NA
	II	2.508(2.179 ~ 2.889)	12.795(< 0.01)	1.419(1.161 ~ 1.734)	3.415(< 0.01)
	III	6.391(5.608 ~ 7.282)	27.831(< 0.01)	3.68(2.914 ~ 4.648)	10.942(< 0.01)
	IV	26.820(23.538 ~ 30.560)	49.387(< 0.01)	9.704(7.634 ~ 12.335)	18.566(< 0.01)
Tstage	T1	Ref	NA	Ref	NA
	T2	1.056(0.875 ~ 1.273)	0.569((0.57)	1.14(0.942 ~ 1.378)	1.345(0.18)
	T3	3.311(2.841 ~ 3.858)	15.330(< 0.01)	1.759(1.445 ~ 2.141)	5.626(< 0.01)
	T4	8.809(7.549 ~ 10.278)	27.639(< 0.01)	3.029(2.48 ~ 3.7)	10.866(< 0.01)
Nstage	N0	Ref	NA	Ref	NA
	N1	3.075(2.854 ~ 3.314)	29.486(< 0.01)	0.74(0.58 ~ 0.945)	-2.415(0.02)
	N2	6.890(6.428 ~ 7.386)	54.482(< 0.01)	0.792(0.585 ~ 1.073)	-1.504(0.13)
Surgery	Surg1	Ref	NA	Ref	NA
	Surg2	0.936(0.866 ~ 1.012)	-1.657(0.098)	1.047(0.964 ~ 1.136)	1.090(0.28)
	Surg3	4.172(3.489 ~ 4.988)	15.662(< 0.01)	2.252(1.56 ~ 3.251)	4.333(< 0.01)
	Surg4	1.280(1.084 ~ 1.510)	2.920(0.003)	0.942(0.789 ~ 1.124)	-0.664(0.51)
LNSur	4 ~	Ref	NA	Ref	NA
	1 ~ 3	1.884(1.637 ~ 2.169)	8.823(< 0.01)	0.927(0.705 ~ 1.219)	-0.54(0.59)
	None	1.400(1.164 ~ 1.680)	3.590(< 0.01)	1.252(1.021 ~ 1.535)	2.161(0.03)
SurgOth	None	Ref	NA	Ref	NA
	Yes	2.431(2.243 ~ 2.634)	21.678(< 0.01)	0.956(0.873 ~ 1.048)	-0.954(0.34)
Radiation	None	Ref	NA	Ref	NA
	Yes	2.219(1.890 ~ 2.605)	9.749(< 0.01)	1.167(0.968 ~ 1.407)	1.624(0.10)
Chemotherapy	None	Ref	NA	Ref	NA
	Yes	0.455(0.431 ~ 0.482)	-27.591(< 0.01)	1.554(1.444 ~ 1.672)	11.774(< 0.01)
Deposits	None	Ref	NA	Ref	NA
	Yes	1.586(1.479 ~ 1.702)	18.878(< 0.01)	1.179(1.093 ~ 1.272)	4.253(< 0.01)
LnPositive	No	Ref	NA	Ref	NA
	1 ~ 3	3.025(2.801 ~ 3.268)	28.119(< 0.01)	1.311(1.044 ~ 1.646)	2.335(0.02)
	4 ~ 6	4.451(4.998 ~ 5.945)	38.318(< 0.01)	1.714(1.276 ~ 2.3)	3.584(< 0.01)
	> 7	8.537(7.909 ~ 9.214)	55.064(< 0.01)	2.234(1.681 ~ 2.971)	5.534(< 0.01)
Bone	None	Ref	NA	Ref	NA
	Yes	6.977(5.267 ~ 9.242)	13.544(< 0.01)	1.23(0.879 ~ 1.722)	1.208(0.23)
Brain	None	Ref	NA	Ref	NA
	Yes	8.483(5.457 ~ 13.186)	9.500(< 0.01)	1.521(0.827 ~ 2.797)	1.35(0.18)
Liver	None	Ref	NA	Ref	NA
	Yes	6.956(6.541 ~ 7.398)	61.225(< 0.01)	1.446(1.302 ~ 1.605)	6.898(< 0.01)
Lung	None	Ref	NA	Ref	NA
	Yes	5.844(5.281 ~ 6.468)	34.124(< 0.01)	1.206(1.064 ~ 1.367)	2.924(< 0.01)
Size	< 3 cm	Ref	NA	Ref	NA
	3 ~ 5 cm	1.088(1.001 ~ 1.173)	2.186(0.03)	0.979(0.902 ~ 1.062)	-0.514(0.61)
	> 5 cm	1.172(1.096 ~ 1.254)	4.621(< 0.01)	0.959(0.893 ~ 1.029)	-1.158(0.25)

### Validation of the model

**Fig. 2:**
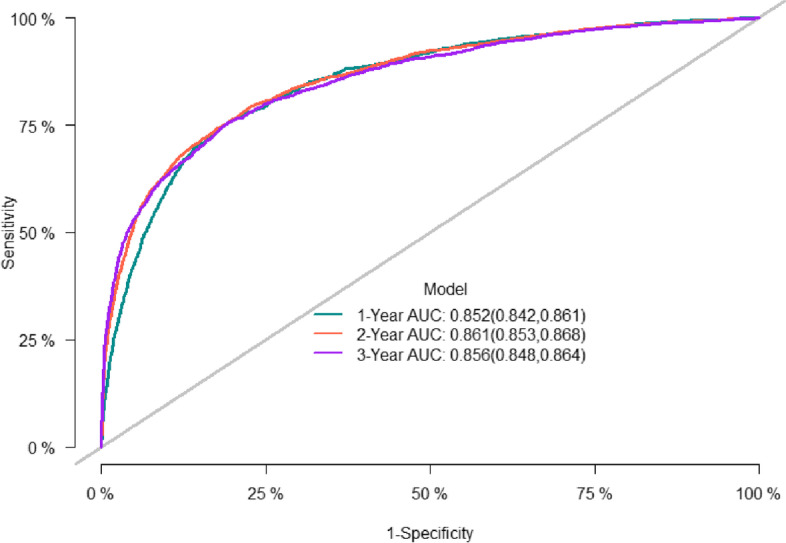
The AUC for OS of 1-, 3- and 5-year of training cohort

**Fig. 3:**
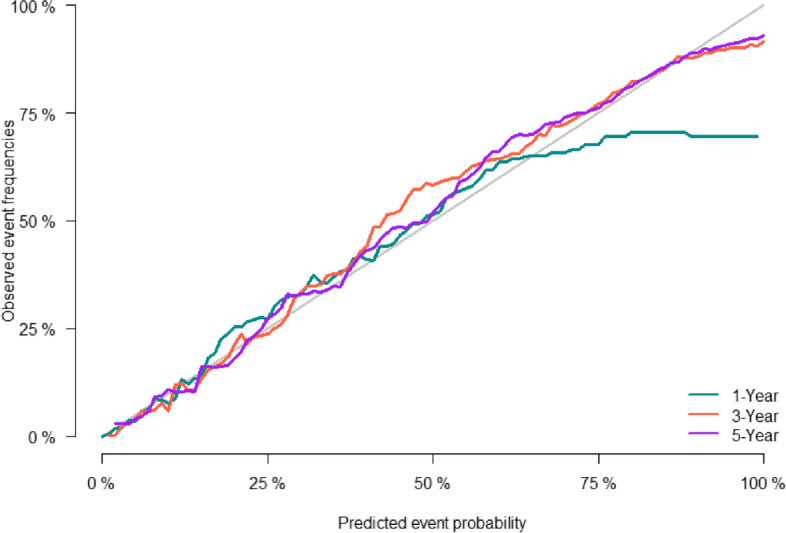
Calibration curves of nomogramfor 1-, 3-, and 5-year CSS in training cohort

**Fig. 4:**
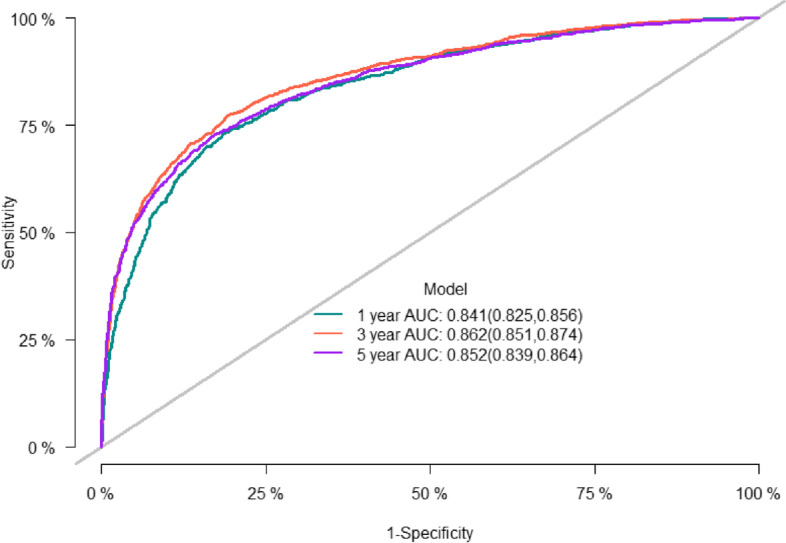
The AUC for OS of 1-, 3- and 5-year of validation cohort

**Fig. 5:**
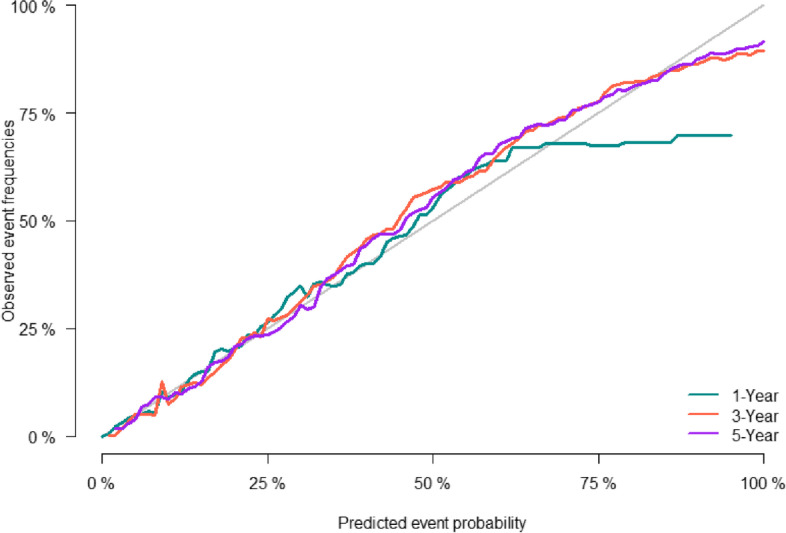
Calibration curves of nomogramfor 1-, 3-, and 5-year CSS in validation cohort

The predictive accuracy of the nomogram was evaluated using the C-index and the area under the RCO curve (AUC). The calibration performance of the nomogram was assessed using the calibration curve. In the training set, the C-index of the model was 0.848 (se:0.0009). The AUC of 1-, 3-, and 5-year survival was 0.852 (95% CI [0.842, 0.861], 0.861 (95% CI [0.853, 0.868]), and 0.856 (95% CI [0.848, 0.864]), respectively (Figure 2), indicating that the model performed well regarding risk prediction and the calibration curve showed that the predicted probability was in good agreement with the observed one (Figure 3). In the validation set, the C-index of the model was 0.847 (se:0.0015), and the AUC at 1-, 3-, and 5-year survival was 0.841 (95% CI [0.825, 0.856]), 0.862 (95% CI [0.851, 0.874]), and 0.852 (95CI [0.839, 0.864], respectively (Figure 4), and the calibration curve showed that the predicted probability was in good agreement with the observed one (Figure 5). Taken together, the nomogram performed well regarding prediction and calibration.

### Comparison of prediction with Cox proportional hazard model and competing risk model

Traditional approaches to survival analysis such as Cox proportional hazard model are used to estimate the probability of one event over time, in which the occurrence of one type of death will prevent the occurrence of the death from other factors. However, in our study, CC-specific death and death from other factors are competing events. Patients dying from non-cancer-related causes accounted for 36.20% of the total deaths. In this scenario, the use of Cox model to analyze competing event data tends to overestimate the mortality in CC patients. In the present study, the predicted probability of mortality at 1 year, 3 years, and 5 years among CC patients estimated using the classical Cox model was 12.98%, 28.37%, and 35.06%, respectively, and that using the competing risk model was 10.8%, 23.59%, and 29.03%, respectively, demonstrating a relatively significant difference between these two models in risk prediction. Specifically, the mortality rate estimated by traditional COX survival analysis was higher than that estimated by the competitive risk model (Table3).

**Table 3:** Cumulative specific mortality at different time points generated using survival analysis and competing hazards models

Time points(months)	Classical Cox proportional hazard model to predict risk of death	Competing risk model	
		cause-specific death	death from other factors
12	12.98	10.8	3.78
24	22.09	18.38	6.58
36	28.37	23.59	9.22
48	32.37	26.88	11.83
60	35.06	29.03	14.46
72	37.03	30.57	17.09
84	38.36	31.58	19.9
96	39.57	32.46	22.51
105	40.51	33.13	24.58

## Discussion

Colorectal cancer is common worldwide and attracts much attention. An estimated 1.2 million people have diagnosed with colorectal cancer annually and over 0.6 million people die from it every year [23, 24]. Recent research has shown that the increase in LCC and primary colon cancer is the largest. Lower survival is seen in patients with RCC than in those with LCC. Patients with CC have the poorest prognosis. In this sense, it is significant to determine variables to accurately predict the survival and prognosis of CC patients since personalized treatment is laid greater stress nowadays.

Standard methods for survival analysis such as the Kaplan–Meier curve and Cox proportional hazards models evaluate time-to-event probabilities. However, these methods tend to produce inaccurate estimates when competing risks exist. Thus, we constructed a nomogram based on clinical data from the SEER database to predict the cause-specific mortality among CC patients. This nomogram evaluated 14 risk factors including tumor pathological classification, tumor grade (degree of differentiation), AJCC stage, T stage, surgery type, lymph node dissection, chemotherapy, tumor deposits, lymph node metastasis, liver metastasis, lung metastasis, etc. It showed good prediction ability in both training and validation datasets.

In the present study, the AJCC stage was the best predictive variable, followed by the T stage. In previous studies, factors including age, race, tumor grade, tumor size, AJCC stage, and surgical status have been identified as independent risk factors for the prognosis of CC patients [25], and this was confirmed by the present research. Historical studies that extracted clinical information from the SEER database to construct Cox proportional hazards models for survival analysis found that race was an independent risk factor for the prognosis of CC patients, and compared with other races, the White had a higher risk of poorer prognosis [11]. However, the present study based on a competing risk model did not support this finding. This may be explained by the biased estimates attributable to the effects of competing risk events. What’s more, we found that CC patients with larger tumor sizes may have a poorer prognosis. However, a multi-center trial in Iran revealed no relevance between tumor size and prognosis [26]. The deviation between the COX regression model and variable effect estimation is a possible reason. On the other hand, the application of the findings in previous studies to the general population is also limited by sample size.

Our research has several limitations. First, the data from the SEER database for statistical analysis featured a short follow-up duration. Second, the nature of this retrospective study makes it difficult to eliminate selection bias. Third, the prognosis of CC patients in our analysis may be affected by patients’ lifestyle, genotype, and other factors, but the data on these factors cannot be obtained from the SEER database, thus related studies are required for further investigation.

## Conclusion

To sum up, this study established a competitive risk model based on clinical data from the SEER database to evaluate the predictor variables for the prognosis of CC patients. Our findings will help clinicians have a better understanding of CC so that they can make appropriate decisions for patients using personalized cancer treatments.

## Data Availability

The original contributions presented in the study are included in the article, further inquiries can be directed to the corresponding author. (extract the data in the SEER database: https://seer.cancer.gov/).
